# Probiotic lactobacilli as a promising strategy to ameliorate disorders associated with intestinal inflammation induced by a non-steroidal anti-inflammatory drug

**DOI:** 10.1038/s41598-020-80482-z

**Published:** 2021-01-12

**Authors:** María José Martínez Monteros, Carolina Maldonado Galdeano, María Florencia Balcells, Ricardo Weill, Juan Andrés De Paula, Gabriela Perdigón, Silvia Inés Cazorla

**Affiliations:** 1 Laboratorio de Inmunología, Centro de Referencia Para Lactobacilos (CERELA-CONICET), San Miguel de Tucumán, Chacabuco 145 - (T4000ILC), Tucumán, Argentina; 2 Cátedra de Inmunología, Facultad de Bioquímica, Química y Farmacia, Universidad Nacional de Tucumán, Tucumán, Argentina; 3 Universidad ISALUD, Buenos Aires, Argentina; 4 Servicio de Gastroenterología, Hospital Italiano, Buenos Aires, Argentina

**Keywords:** Intestinal diseases, Mucosal immunology, Microbiology

## Abstract

Damage to the small intestine caused by non-steroidal anti-inflammatory drugs (NSAIDs) occurs more frequently than in the upper gastrointestinal tract, is more difficult to diagnose and no effective treatments exist. Hence, we investigated whether probiotics can control the onset of this severe condition in a murine model of intestinal inflammation induced by the NSAID, indomethacin. Probiotic supplementation to mice reduce the body weight loss, anemia, shortening of the small intestine, cell infiltration into the intestinal tissue and the loss of Paneth and Goblet cells associated with intestinal inflammation. Furthermore, a high antimicrobial activity in the intestinal fluids of mice fed with probiotics compared to animals on a conventional diet was elicited against several pathogens. Interestingly, probiotics dampened the oxidative stress and several local and systemic markers of an inflammatory process, as well as increased the secretion of IL-10 by regulatory T cells. Even more importantly, probiotics induced important changes in the large intestine microbiota characterized by an increase in anaerobes and lactobacilli, and a significant decrease in total enterobacteria. We conclude that oral probiotic supplementation in NSAID-induced inflammation increases intestinal antimicrobial activity and reinforces the intestinal epithelial barrier in order to avoid pathogens and commensal invasion and maintain intestinal homeostasis.

## Introduction

The gastrointestinal (GI) tract is an organ system whereby a balance between the epithelial cells, the immune system and the resident microbiota play a crucial role for maintaining intestinal homeostasis. The physical barriers include a single layer of epithelial cells, their intercellular tight junctions, and the mucus that covers the epithelial surface. Mucins, the major components of the mucus layer, are secreted by Goblet cells that are interspersed among enterocytes throughout the epithelium^1^. The epithelial layer not only serves as a passive physical barrier between the host and the intestinal microbiota. Intestinal epithelial cells (IECs) sense external or endogenous danger signals and mount a robust immune response^2^. When the barrier function is impaired, the contact of the intestinal mucosa with the resident colonic bacteria leads to intestinal disorders.

Moreover, Paneth cells, located at the bottom of the intestinal crypts, are responsible for the secretion of a diverse arsenal of antimicrobial peptides (AMPs) such as lysozymes, secretory phospholipase A2, defensins, defensin-like peptides and cathelicidins, which have high antimicrobial activity. This array of AMPs limits the invasion and adherence of pathogenic and commensal bacteria by disrupting the integrity of the bacterial cell membrane or wall^3^. In the last years, increasing evidence suggests that an impaired or defective production of AMPs is already implicated in Crohn’s disease^4–7^. Once the inflammatory response has been initiated, IECs play an active role in the resolution of inflammation by secreting anti-inflammatory mediators that generally inhibit neutrophil function.

Inflammatory bowel disease (IBD) is the collective name for a group of chronic inflammatory gastrointestinal disorders, including ulcerative colitis, Crohn's disease (which constitutes the vast majority of the cases), and other less frequent conditions that share features of both disorders (non-classifiable inflammatory bowel disease and indeterminate colitis).

Although the mechanisms underlying the etiology of IBD remain largely unknown, they involve a complex interaction of genetic and environmental factors, in a background of oxidative stress together with a dysregulation of the enteric immune system and alterations in the intestinal microbiome^8–10^. Drugs that have been linked to cause or worsen IBD-like conditions include isotretinoin, antibiotics, oral contraceptives, mycophenolate mofetil, etanercept, ipilimumab, rituximab, sodium phosphate and non-steroidal anti- inflammatory drugs (NSAIDs)^11^. Is attractive to suggest that the anti-inflammatory actions of NSAIDs are due to inhibition of cyclooxygenase (COX) COX-2, whereas the unwanted side-effects, such as irritation of the stomach lining, are due to inhibition of COX-1^12^. NSAIDs long-term administration often causes gastrointestinal lesions characterized by a breaking down in epithelial tight junctions, reduction on mucus production, abnormal passage of luminal antigens and intestinal bacteria, and increases in the local and systemic inflammatory response^13,14^.

No certified clinical strategies for preventing NSAID-induced small intestinal injury is currently available. The use of anti-TNFα and other cytokine monoclonal antibodies have been introduced both for inducing remission and for maintenance therapy in refractory forms of the disease. However, the persistent and marked blockage of TNF-α bioactivity may have a detrimental effect on acute intestinal inflammation^15,16^.

Probiotics have emerged as an important tool for this devastating disease as they can modulate immune disturbances and intestinal dysbiosis^17–20^. Probiotic lactobacilli adhering to mammalian tissues can establish an upheld crosstalk with the host reinforcing the intestinal barrier and leading to the competitive exclusion of pathogens^21,22^.

We have previously demonstrated that oral lactobacillus administration increases the Paneth cells and the intestinal antimicrobial activity in healthy mice^23^. Here, we explore two probiotic lactic acid bacteria (*Lactobacillus casei* CRL 431 and *Lactobacillus paracasei* CNCM I- 1518) as promising weapons to counteract the intestinal inflammation induced by NSAIDs. We will focus here on the effect of orally administered probiotics as enhancers of the antimicrobial activity along the gastrointestinal tract and modulators of the intestinal microbiota, considering resident and pathogenic bacteria encountered at these specific sites involved in the pathogenesis of intestinal inflammation.

## Results

### Probiotic bacteria ameliorate clinical signs of intestinal inflammation induced by indomethacin in mice

**Figure 1:**
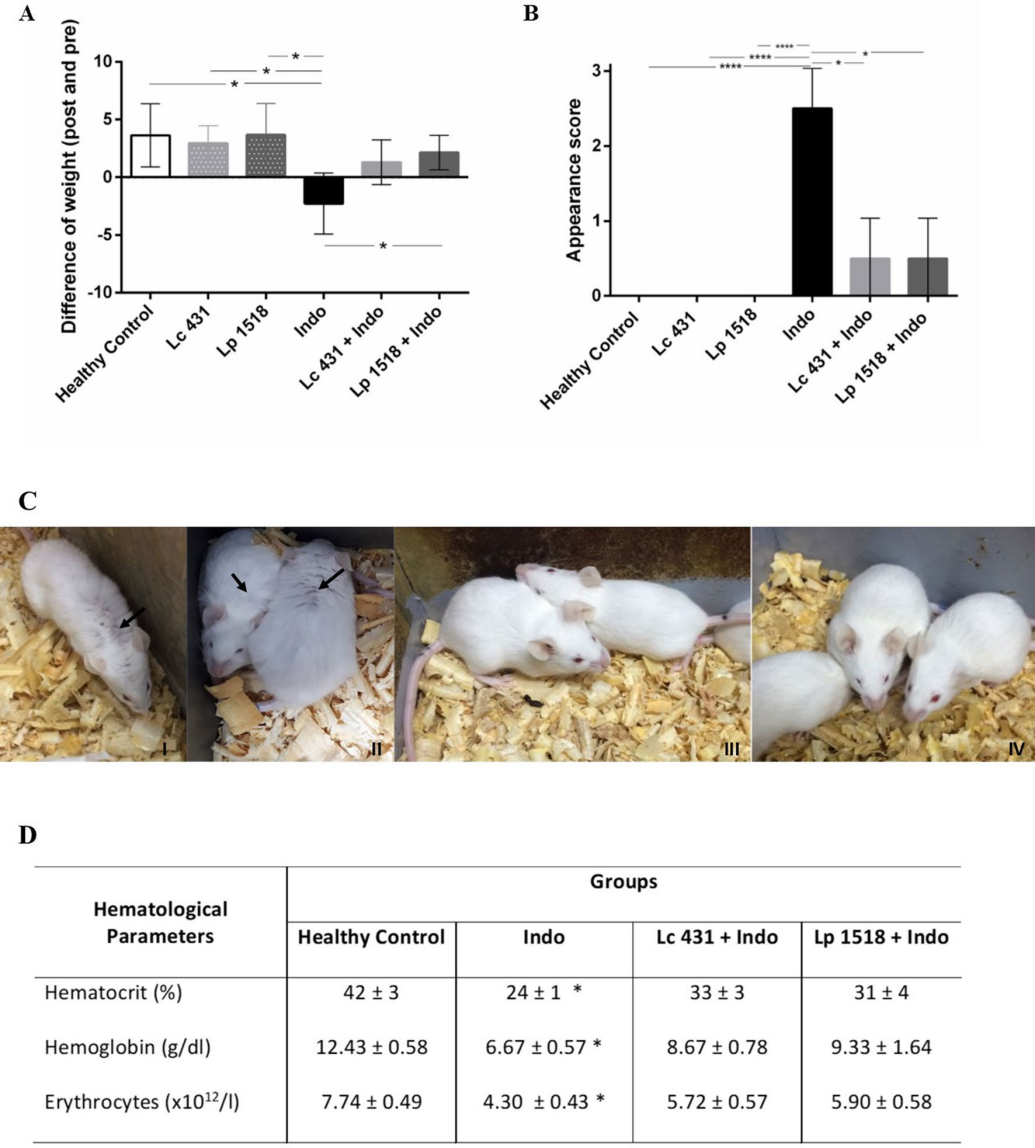
Clinical features of intestinal inflammation in animals. Balb/c mice supplemented with *Lactobacillus casei* CRL 431 (Lc 431), *Lactobacillus paracasei* CNCM I-1518 (Lp 1518), or water, for 7 and 5 days, received indomethacin injections on the last two days. Additional controls fed with Lc 431, Lp 1518 or water, received 2 PBS injections. (**A**) Body weight differences after and before the indomethacin injection. (**B**) Appearance score was evaluated according to Shrum et al.^51^. The line at the top of the bars indicates the comparison between the two groups, *p < 0.05. (**C**) Photography of mice. Panels I- and II- show important patches of piloerected hair pointed by black arrows, in the Indo group. By contrast, the coat is smooth in Lc 431 + Indo and Lp 1518 + Indo groups (Panel III- and IV-, respectively). (**D**) Evaluation of red blood cells on the day mice were euthanized. Stars show the comparison to the healthy control group. *p < 0.05.

Animals fed with *L. casei* CRL 431 (Lc 431), *L. paracasei* CNCM I-1518 (Lp 1518), or water, for 7 and 5 days, respectively, received two indomethacin injections in the last two days of probiotic supplementation. Weight loss being one of the clinical features of intestinal inflammation, was registered before and after the indomethacin injection. Importantly, we observed a significant body weight loss after indomethacin treatment in animals receiving a conventional diet (Indo group: -2.27) compared to healthy controls (*p* < 0.05). By contrast, mice with indomethacin-induced inflammation supplemented with the probiotics were even able to increase their body weight compared to those values registered before the injection of indomethacin (1.30 ± 0.97 and 2.14 ± 0.52, for Lc 431 + IBD and Lp 1518 + IBD, respectively) (Fig. 1A). During the time analyzed none of the animals had diarrhea.

The physical appearance of mice was also evaluated, observing piloerection and a swollen appearance in those animals receiving the indomethacin injection in the absence of probiotic supplementation (Indo group). By contrast, Lc 431 + Indo and Lp 1518 + Indo animals, only showed small patches of piloerected hair and normal appearance (*p* < 0.05) (Fig. 1B,C). It is important to mention that those littermate animals receiving the oral supplementation with Lc 431 and Lp 1518, without the indomethacin injection, showed significant differences in their body weight and appearance with respect to healthy control animals on a conventional diet at the time analyzed (Fig. 1A,B). Since, in the absence of indomethacin-induced intestinal inflammation, the immune response elicited in mice upon the probiotic supplementation (Lc 431 and Lp1518 groups) has been extensively described by us, this group was not included in the following studies.

We then analyzed the presence of anemia, a clinical sign that is frequently observed in patients suffering from chronic inflammatory disorders. No differences were observed in hematocrit values before the indomethacin injections in the different groups (45 ± 1.56, 43 ± 1.44, 44 ± 1 0.5 and 43 ± 1.3% for healthy controls; Indo; Lc 431 + Indo and Lp 1518 + Indo; respectively) (data not shown). By contrast, we observed a significant decrease in hematocrit, hemoglobin values and red blood cell counts in Indo animals compared to healthy controls (*p* < 0.05) after induced-intestinal inflammation. Importantly, these parameters were only slightly modified in Lc 431 + Indo and Lp 1518 + Indo, being not statistically different from healthy controls (Fig. 1D).

**Figure 2:**
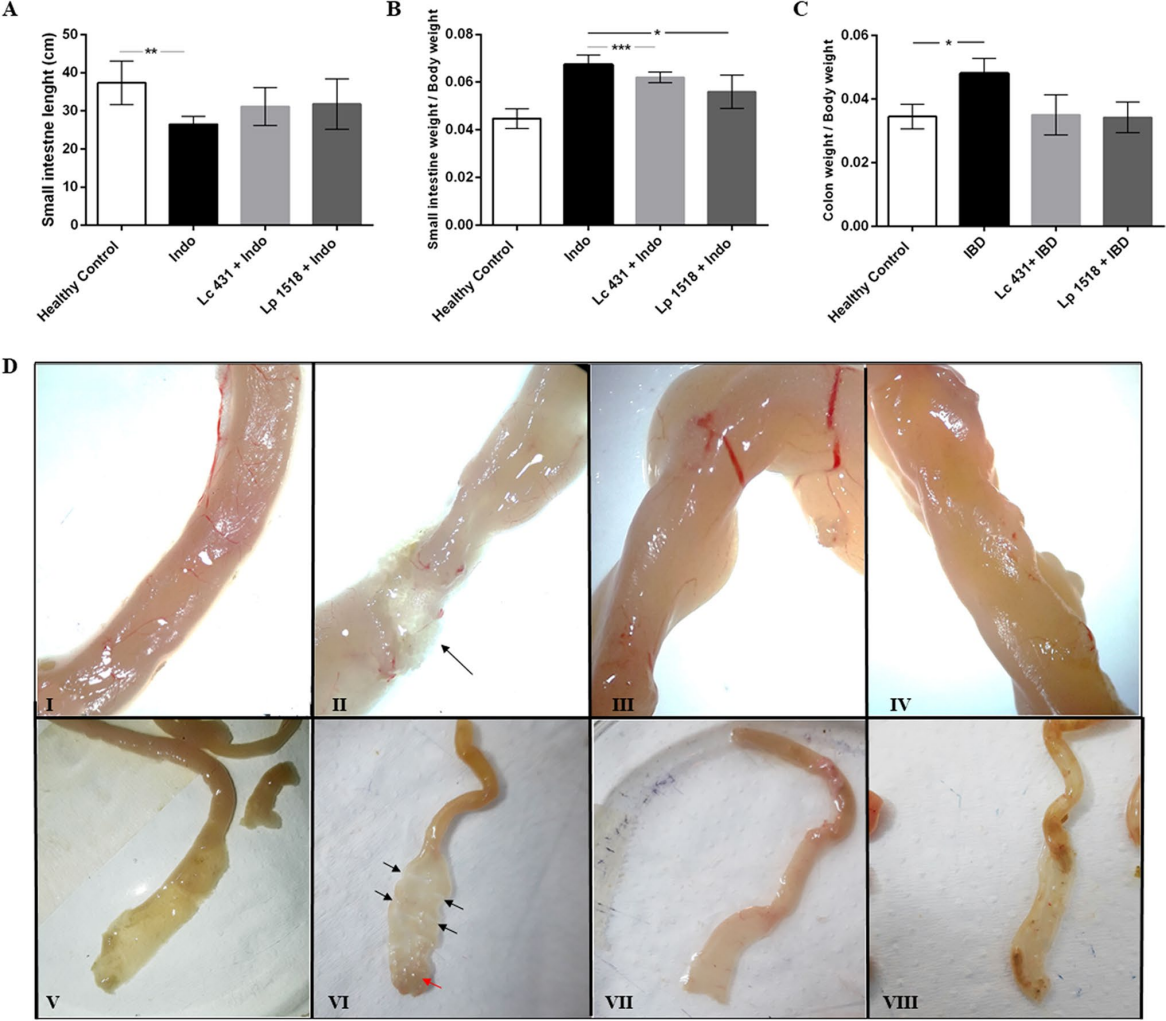
Intestinal damage in mice treated with indomethacin and supplemented with probiotics. Balb/c mice supplemented with *Lactobacillus casei* CRL 431 (Lc 431), *Lactobacillus paracasei* CNCM I-1518 (Lp 1518), or water, for 7 and 5 days, received indomethacin on the last two days. On day 8 mice were euthanized and the following parameters were evaluated: (**A**) small intestine length; (**B**) the ratio between the weight of the small intestine and the body of each animal; and (**C**) the ratio between the weight of each large intestine and the body of the animal. The line at the top of the bars indicates the comparison between the two groups. *p < 0.05, **p < 0.01, ***p < 0.001. (**D**) Representative photography of the small intestine of: Panel I and V- Healthy control; Panel II and VI- Indo; Panel III and VII- Lc 431 + Indo; and Panel IV and VIII- Lp 1518 + Indo mice, revealing the presence of ulcers (black arrows) and a mucosa with granular appearance (red arrows) in mice on a conventional diet treated with indomethacin. I-IV images were taken under observation with a Leica ES2 Stereo Microscope with 10 × High Eyepoint. V-VIII are images of the intestine after cutting them longitudinally.

Mice were euthanized on day 8 and samples of small and large intestine were taken. We observed a significant shortening of the small intestine in Indo animals leading to a substantial shrinkage of the organ in comparison to healthy littermate controls (*p* < 0.01 and *p* < 0.005, respectively). These modifications, as well as those observed in the large intestine, were ameliorated in animals with Indo receiving the oral probiotic supplementation (Lc 431 + Indo and Lp 1518 + Indo) (Fig. 2B–D).

The small intestine was then submitted to careful macroscopic observation, showing the presence of ulcers and a multitude of small points of light which conferred a granular appearance to the mucosa in the Indo groups (Fig. 2D). By contrast, no damage was observed in Lc 431 + Indo and Lp 1518 + Indo nor in the healthy controls for the macroscopic analysis.

**Figure 3:**
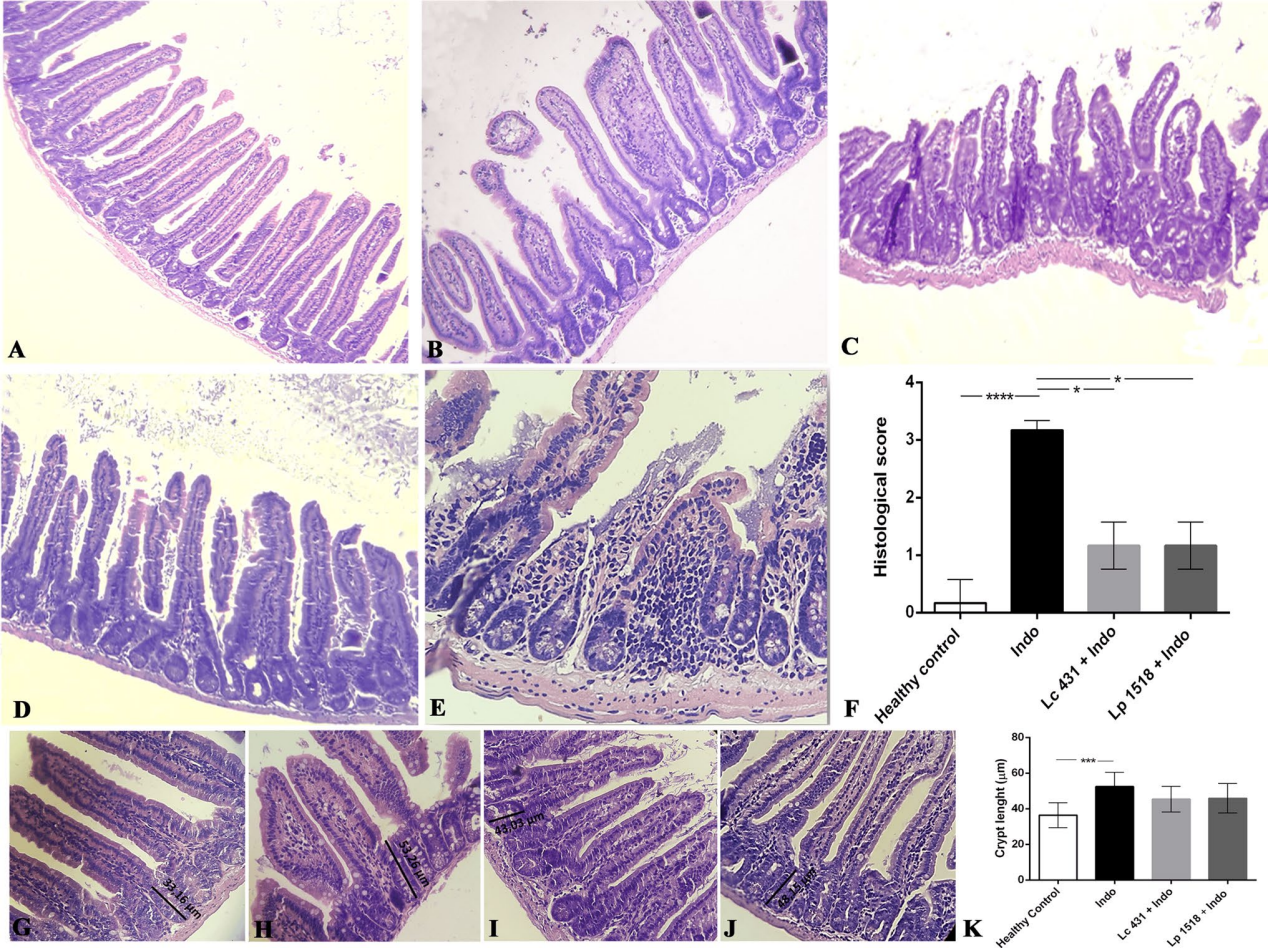
Hematoxylin and eosin staining of small intestine tissue section. Balb/c mice supplemented with *L. casei* CRL 431 (Lc 431), *L. paracasei* CNCM I-1518 (Lp 1518), or water, for 7 and 5 days, received indomethacin injections on the last two days. On day 8 mice were euthanized and samples of the small intestine were taken for histological analysis. Representative micrographs of: (**A**,**G**) Healthy control; (**B**,**E**,**H**) mice on a conventional diet treated with indomethacin; (**C**,**I**) Lc 431 + Indo; and (**D**,**J**) Lp 1518 + Indo. (**D**) Inflammatory cells infiltrated in indomethacin-treated mice on a conventional diet. (**F**) Inflammation was qualitatively evaluated according to the number and spread of inflammatory foci from 0 to 4: 0, no leukocytic infiltration; 1, low level of leukocytic infiltration; 2, moderate level of leukocytic infiltration; 3, high level of leukocytic infiltration; and 4, transmural infiltration^54^. *p < 0.05 ***p < 0.001. (**K**) Length of Lieberkühn crypt in the small intestine of experimental mice. Results (mean ± S.E.M.) are representative of three independent experiments. The line at the top of the bars indicates comparison between the two groups. ***p < 0.01. Magnification: (A-D) 100X, (E, G-J) 400X. Lines in panels G, H, I and J mean: 33.16, 53.26, 43.03 and 48.15 µm, respectively.

We then performed the histological analysis of the small intestine to further examine the effects of probiotic consumption on an intestinal inflammatory process. A normal architecture of the small intestine was shown in healthy control mice (Fig. 3A), by contrast, we observed in the hematoxylin–eosin staining of tissue section that the administration of indomethacin caused overt damage to intestinal villi. Irregular villi with variable diameters along the depth of single crypts of dilated crypts, villi that did not run parallel, and a marked inflammatory cell infiltration were observed (Fig. 3B,E). By contrast, intestinal villi exhibited a relatively normal structure without significant alterations in the mucosal architecture of mice with intestinal inflammation receiving probiotic supplementation (Lc 431 + Indo and Lp 1518 + Indo) (Fig. 3C,D). Additionally, these mice exhibited Lieberkühn crypts with similar length to those observed in healthy control animals, while mice with intestinal inflammation on a conventional diet showed an important hyperplasia of the crypt compared to healthy control mice (*p* < 0.001) (Fig. 3G–J).

### Oral probiotic supplementation in indomethacin-induced intestinal inflammation reinforces the intestinal barrier by increasing antimicrobial activity and Paneth cells

**Figure 4:**
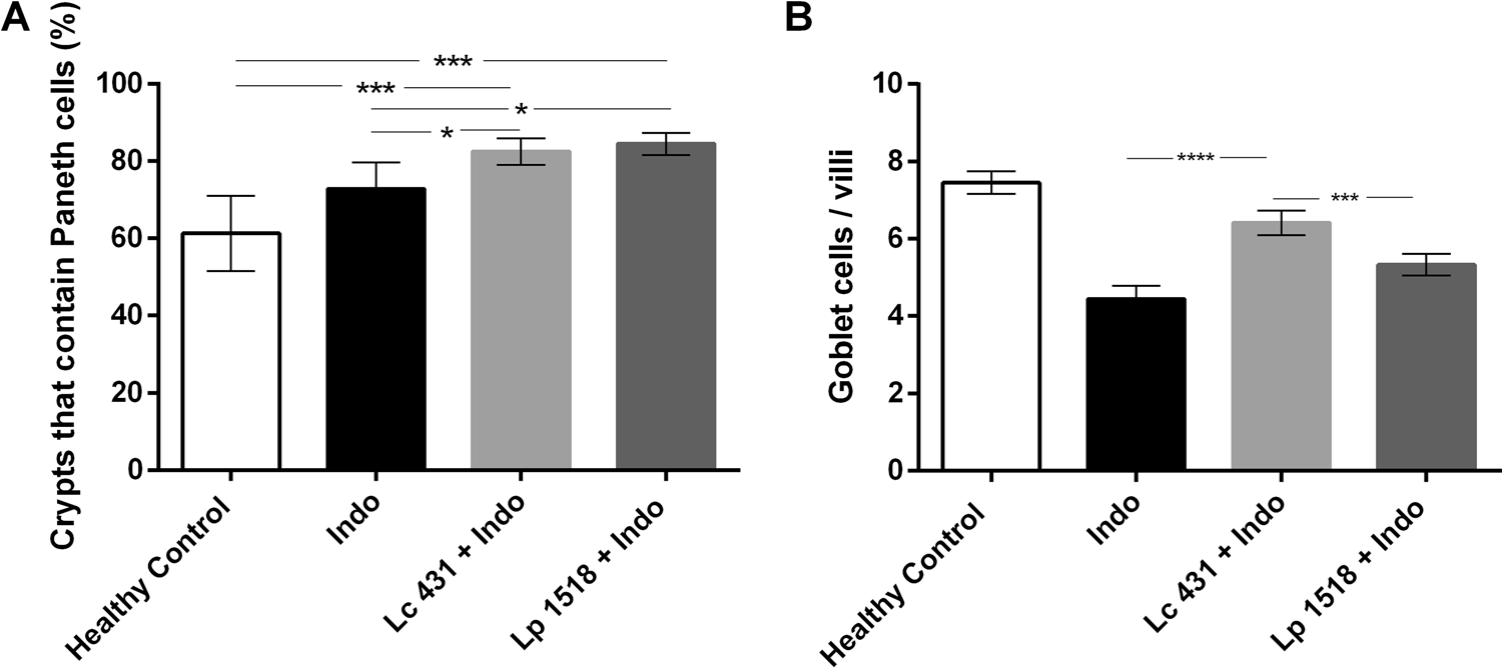
Mouse intestinal secretory cells in indomethacin-induced intestinal inflammation. Tissue sections of Balb/c mice orally supplemented with *Lactobacillus casei* CRL 431 (Lc 431), *Lactobacillus paracasei* CNCM I-1518 (Lp 1518), or water, for 7 and 5 days and receiving 2 indomethacin injections were stained with hematoxylin and eosin and examined by light microscopy. A blind histological test of the small intestine was performed by analyzing 5 slices of each organ and the percentages of (**A**) crypts with positive Paneth cells and (**B**) Goblet cells per villus were determined. Results were expressed as the number of Goblet cells per intestinal villus. The column bar shows a semi-quantitative evaluation for each group. Each bar represents the group mean ± S.E.M. Results are representative of three independent experiments. The line at the top of the bars indicates comparison between the two groups. *p < 0.05, **p < 0.01, ***p < 0.001, ****p < 0.0001.

Considering indomethacin administration used to impairs the functions of the gut barrier, the number of Paneth and Goblet cells was determined in all the experimental animals. We observed a significant increase in Paneth cells in Lc 431 + Indo and Lp 1518 + Indo compared to the Indo group (*p* < 0.05) (Fig. 4A). Moreover, the number of mucin-secreting Goblet cells was significantly reduced in Indo (4.43 ± 0.33, mean ± S.E.M) compared to healthy controls (*p* < 0.0001). Importantly, oral probiotic supplementation was able to greatly increase the number of Goblet cells (6.41 ± 0.31 and 5.32 ± 0.28, mean ± S.E.M; for Lc 431 + Indo and Lp 1518 + Indo groups, respectively) (Fig. 4B).

No bacterial translocation from the commensal microbiota to spleen or liver was observed in any of the experimental animals. Those organs appeared to be sterile after serial dilution plating of the organs on agar medium.

**Figure 5:**
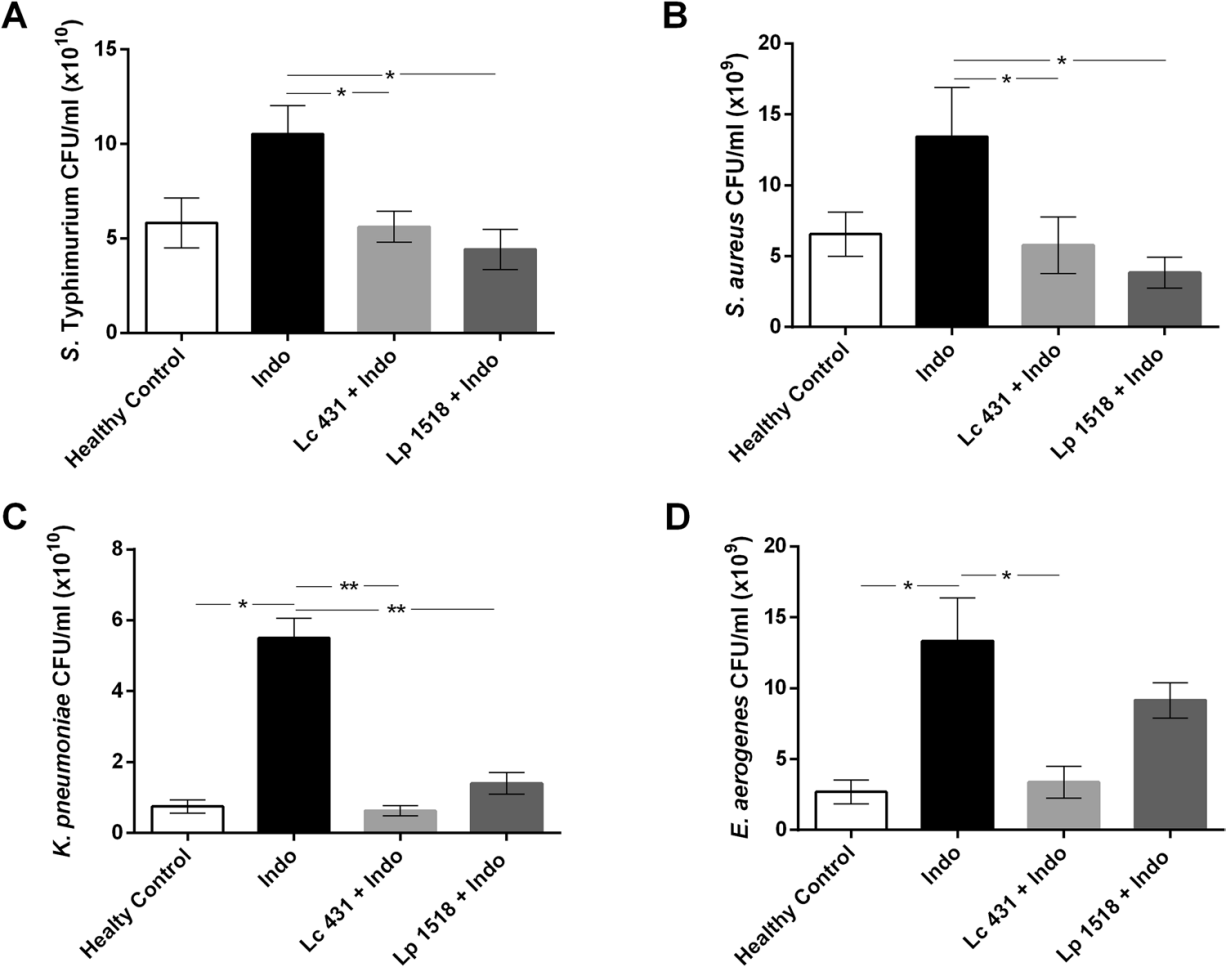
Antimicrobial activity of intestinal fluids of indomethacin-treated mice. *S. aureus* (**A**), *S.* Typhimirium (**B**), *K. pneumoniae* (**C**) and *E. aerogenes* (**D**), (10^9^ CFU/ml), were incubated at 37 °C for 2 h in the presence of the intestinal fluids of: healthy control animals, or mice with induced intestinal inflammation receiving a conventional diet or probiotic supplementation (Indo; Lc 431 + Indo and Lp 1518 + Indo groups, respectively). After co-incubation, viable bacteria were determined by plate count agar. A set of serial dilutions were made and samples of each appropriate dilution were spread on top of solidified agar petri plates. Results (mean ± S.E.M) are representative of three independent experiments. Results were expressed as the differences in CFU/ml after and before the incubation of bacteria with the intestinal fluids. The line at the top of the bars indicates comparison between the two groups. *p < 0.05, **p < 0.01.

While considering the resident and pathogenic flora encountered in the intestinal lumen to be involved in the pathogenesis of inflammatory processes along the gastrointestinal tract, the antimicrobial activity of the intestinal fluids in the experimental animals was analyzed. The increase under probiotic supplementation in the number of crypts containing Paneth cells was in accordance with the antimicrobial activity observed in the intestinal fluids of the mice. An altered intestinal antimicrobial activity in the Indo animals was revealed by a change in the growth kinetic of the pathogen, when *Staphylococcus aureus* and *Salmonella enterica serovar* Typhimurium were incubated in the presence of the intestinal fluid of those animals (13.42 ± 3.47 × 10^10^ and 10.56 ± 1.49 × 10^9^ for *S. aureus* and *S.* Typhimurium, respectively, vs 6.55 ± 1.55 × 10^10^ and 5.85 ± 1.32 × 10^9^ for healthy controls). Additionally, a great decrease in the CFU/ml of *S. aureus* and *S.* Typhimurium was observed after their incubation with the intestinal fluids of Lc 431 + Indo and Lp 1518 + Indo, compared to those registered in mice on a conventional diet receiving the indomethacin injection (*p* < 0.05). More interestingly, the CFU of both pathogens in Lc 431 + Indo and Lp 1518 + Indo were similar to those registered in healthy mice controls (Fig. 5A,B).

The antimicrobial activity of the intestinal fluids was also evaluated against multi-resistant bacteria to several antibiotics. It is worth highlighting that a significant decrease in the CFU of *K. pneumonia* and *E*. *aerogenes* by plate count agar was observed after their incubation with the intestinal fluids of probiotic-fed mice (Lc 431 + Indo and Lp 1518 + Indo) compared to the animals on a conventional diet receiving the indomethacin injection (*p* < 0.05, and *p* < 0.01) (Fig. 5C,D).

These results together indicate that the increase in probiotic-induced intestinal antimicrobial activity can contribute to restore the disruption of gut barrier integrity observed in the indomethacin-induced intestinal inflammation, preventing bacterial permanence in the intestinal tract leading to sustained inflammation and tissue damage.

### Oral probiotic supplementation promotes a reduction in the intestinal inflammatory process in indomethacin-induced intestinal inflammation

**Figure 6:**
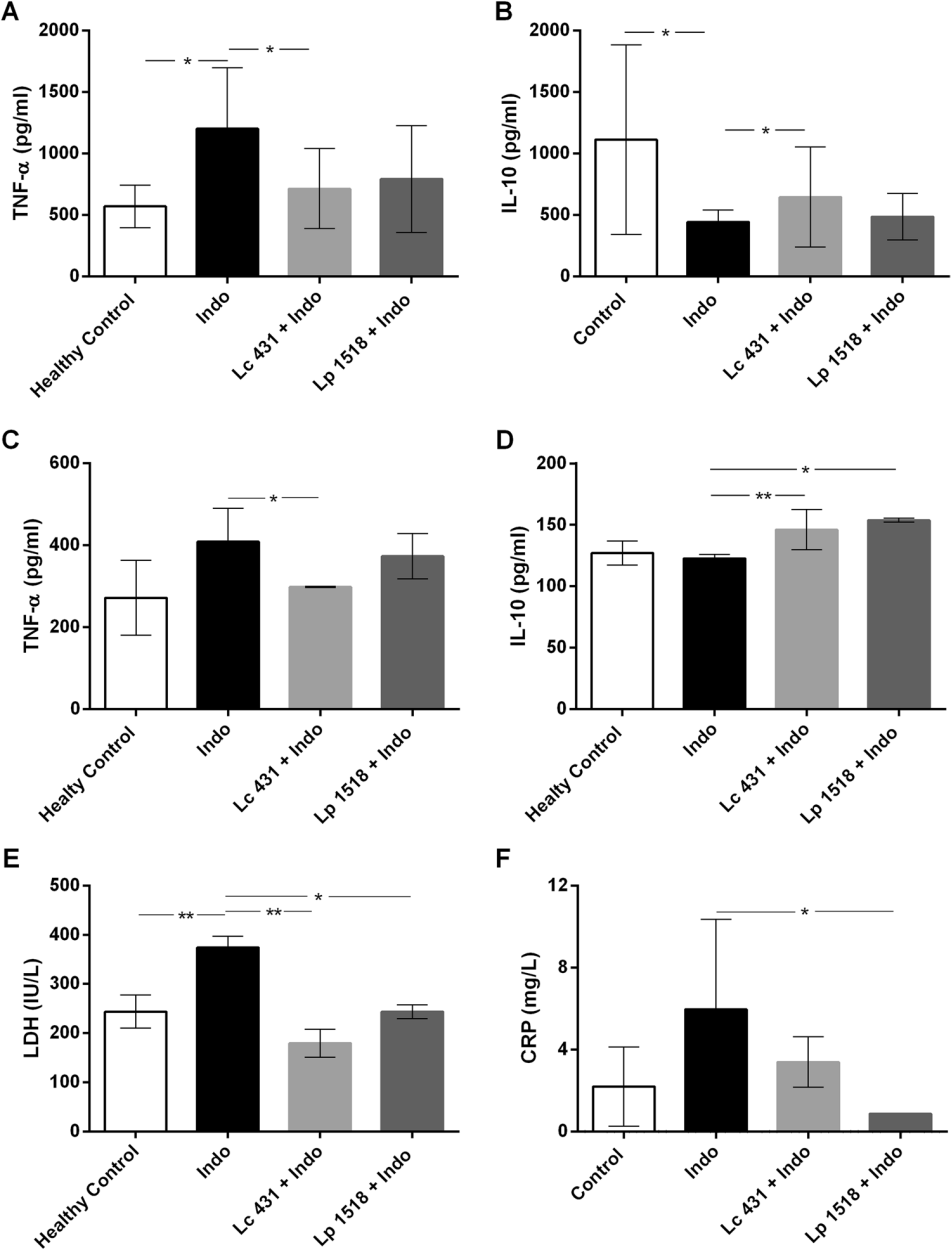
Cytokine secretion in the intestinal microenvironment of indomethacin-treated mice fed probiotics. TNF-α (**A**,**C**) and IL-10 (**B**,**D**) were determined in the (**A**,**B**) intestinal fluids and (**C**,**B**) supernatant of the epithelial cell culture from healthy control mice and animals with an intestine inflammation process receiving a conventional diet (Indo) or oral supplementation with probiotics (Lc 431 + Indo and Lp 1518 + Indo). Samples were assayed in duplicate by Capture ELISA. The results are representative of 3 independent experiments (n = 6). The line at the top of the bars indicates comparison between the 2 groups. *p < 0.05 and ** *p* < 0.01.

One of the mechanisms involved in the pathogenesis and establishment of IBD is a hyper activation of the proinflammatory pathways associated with a defective counter-regulatory mucosal immune response. Accordingly, in the intestinal fluids of Indo mice fed with a conventional diet we observed a significant increase in TNF-α levels as well us low levels of IL-10, compared to healthy mice controls (*p < 0.05) (Fig. 6). Interestingly, animals treated with indomethacin receiving oral probiotic supplementation exhibited lower levels of TNF-α in their intestinal fluids (Lc 431 + Indo: 715 ± 71.09 pg/ml and Lp 1518 + Indo: 793.8 ± 97.07 pg/ml) compared to those littermates on a conventional diet (Indo: 1,202 ± 117.3 pg/ml) (Fig. 6A).

Notably, this dampening in proinflammatory cytokine secretion by intestinal epithelial cells was coupled with an enhancement of IL-10 in Lc 431 + Indo and Lp 1518 + Indo, (**p < 0.01 and *p < 0.05, respectively) in comparison to animals with intestinal inflammation fed on a conventional diet (Fig. 6B,D). These results highlight the role of probiotics to hold on the balance between TNF-α and IL-10; this fact being critical to preserve intestinal homeostasis in indomethacin-induced intestinal inflammation.

As additional markers of an inflammatory process, we measured the serum protein levels that are released into the blood when cells are damaged: lactate dehydrogenase (LDH) and C-reactive protein (CRP), an acute phase reactant. As shown in Fig. 6E indomethacin-treated mice receiving oral probiotic supplementation showed significant lower serum LDH levels compared to those on a conventional diet (**p < 0.01 and *p < 0.05, Lc 431 + Indo and Lp 1518 + Indo, respectively). Similarly, mice with an intestine inflammatory process receiving a conventional diet exhibited higher CRP levels compared to those fed with probiotics (Indo: 5.96 ± 1.96 mg /ml, Lc 431 + Indo: 3.39 ± 0.61 mg/ml and Lp 1518 + Indo:2.37 ± 0.28 mg/ml) (Fig. 6F).

### Probiotic administration in indomethacin-induced intestinal inflammation decreased the overproduction of reactive oxygen species (ROS) and proinflammatory cytokines by macrophages

**Figure 7:**
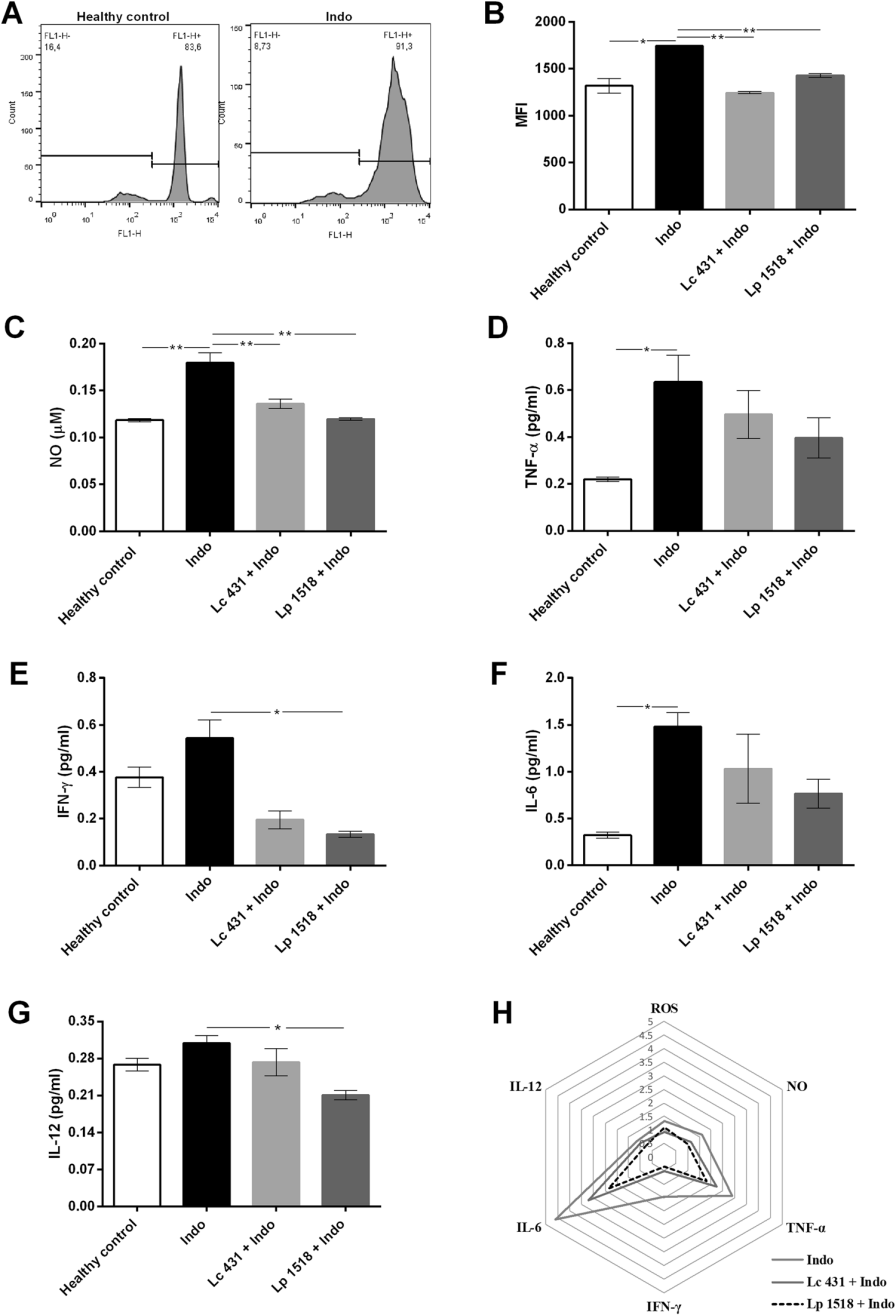
Release of reactive oxygen species and cytokine mediators of intestinal inflammation by macrophages. Balb/c mice supplemented with *L. casei* CRL 431 (Lc 431), *L. paracasei* CNCM I-1518 (Lp 1518), or water, for 7 and 5 days, received indomethacin injections on the last two days. On day 8, mice were euthanized and samples of peritoneal macrophages were taken. ROS production as fluorescence units of oxidized dichlorodihydrofluorescein was detected by flow cytometry and (**A**) representative histograms and (**B**) mean fluorescence intensity (MFI) in experimental animals were shown. Peritoneal macrophages were incubated at 37 °C 5% CO_2_ for 16 h and the supernatant collected for the determination of: (**C**) NO by Griess reagent; (**D**,**E**,**F**,**G)** proinflammatory cytokines by Capture ELISA. Results are expressed as mean ± SEM and are representative of at least three independent experiments. The line at the top of the bars indicates comparison between the 2 groups. *p < 0.05 and ** *p* < 0.01. (**H**) Radar chart plot of inflammatory mediators released by macrophages from the different experimental groups. Results are expressed as the ratio between each group and the healthy control mice.

An overwhelming reactive oxygen and nitrogen species generation has been implicated as mediators of mucosal injury in several inflammatory process. Accordingly, the endogenous generation of ROS by peritoneal macrophages from experimental mice was evaluated using the ROS-dependent conversion of the oxidative-sensitive probe H2DCFDA into the fluorescent dye dichlorofluorescein by flow cytometry. By a previous selection of the peritoneal macrophages in a forward versus side-scatter dot plot, a significant increase in the production of ROS in Indo mice was observed compared to healthy mice controls (*p < 0.05). More interestingly, oral probiotic supplementation in the mouse model of intestinal inflammatory process was able to significantly decrease the ROS production compared to those littermates on a conventional diet (IBD) (**p < 0.01) (Fig. 7A,B).

To further explore the modulation elicited by probiotics in reactive oxygen and nitrogen species, the levels of nitric oxide (NO) were measured at 16 h in the macrophage culture supernatant. In accordance with ROS production, a great reduction in NO levels was observed in Lc 431 + Indo and Lp 1518 + Indo with respect to IBD animals (Fig. 7C).

Finally, the production of macrophage proinflammatory cytokines playing an important role in the development of inflammatory bowel disease was evaluated, finding an important decrease in the release of TNF-α, IL-6, IFN-γ and IL-12 indomethacin-treated mice fed with probiotics compared to those on a conventional diet (Fig. 7D–G). These results together showed that oral probiotic supplementation ameliorates indomethacin-induced inflammatory responses elicited by macrophages mainly through a reduction in ROS and proinflammatory cytokine production and contributes to promoting intestinal homeostasis (Fig. 7H).

### Probiotic supplementation in indomethacin-treated mice promotes intestinal homeostasis

**Figure 8:**
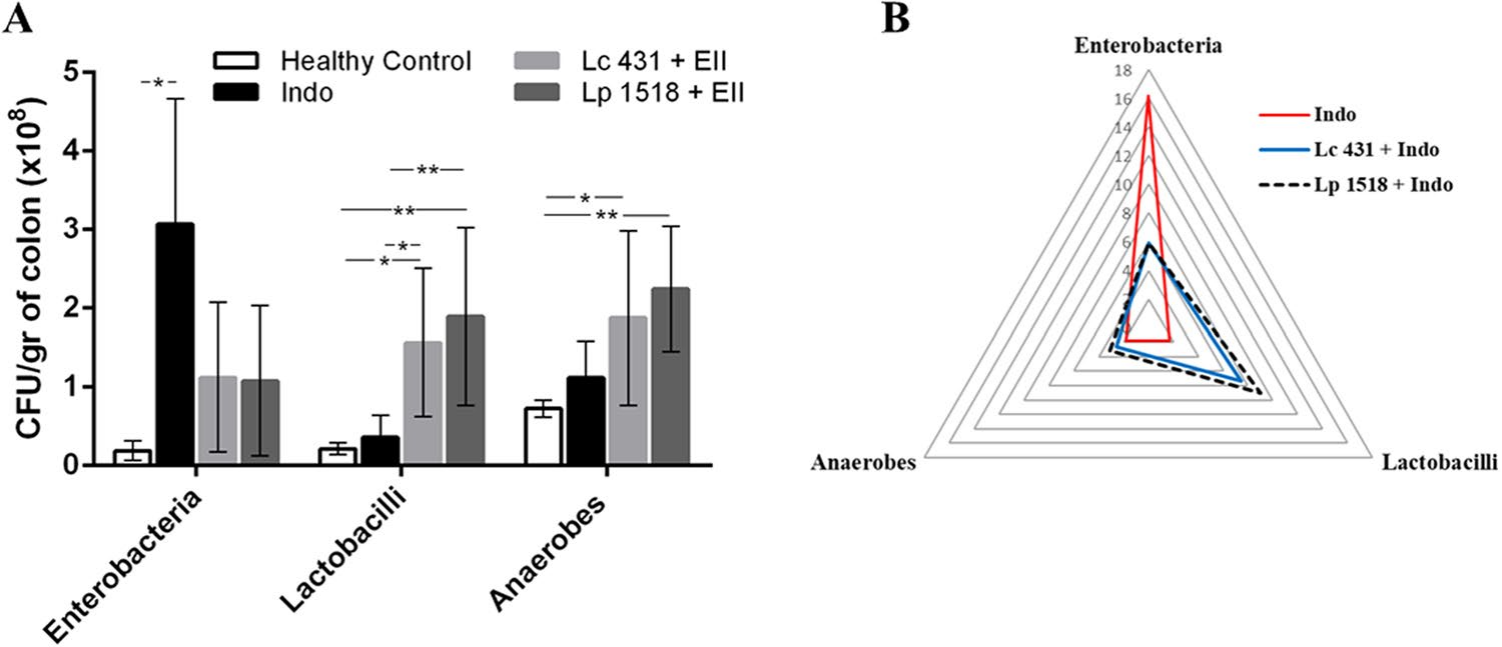
Microbial population in the large intestine. Balb/c mice with an indomethacin-induced intestinal inflammatory process receiving a conventional diet (Indo) or oral probiotic supplementation (Lc 431 + Indo and Lp 1518 + Indo) for 7 and 5 days, respectively. At the end of this time, samples of the large intestine were collected and total anaerobic bacteria, lactobacilli, and enterobacterial population were analyzed by plate count agar. (**A**) Results were expressed as CFU/ml per gram of large intestine (mean ± S.E.M). Three independent experiments were performed. The line at the top of the bars indicates comparison between the two groups. *p < 0.05, **p < 0.01. (**B**) Mean scores of total bacterial population data in the experimental animals. Results are expressed as the ratio/relationship between each group and healthy controls.

Finally, we analyzed the role of the large intestinal microbiota in the onset of intestinal inflammation. Colons of experimental mice were removed, homogenized and placed in agar plate medium specific for different bacteria. As shown in Fig. 8 a significant increase in total enterobacteria in the Indo group was observed compared to healthy mice controls (*p < 0.05). Interestingly, in mice receiving oral probiotic supplementation (Lc 431 + Indo and Lp 1518 + Indo) a slight decrease in total enterobacterial population was observed compared to those mice with intestinal inflammation on a conventional diet. As expected, animals fed with probiotic lactic acid bacteria exhibited higher levels in lactobacillus count. Moreover, a significant increase in total anaerobic bacteria was also observed in animals receiving probiotic lactic acid bacteria supplementation compared to healthy controls (Fig. 8A). These results suggest that in an intestinal inflammatory process the administration of Lc 431 and Lp 1518 promotes the expansion of protective microorganisms and the reduction of detrimental bacteria to maintain intestinal microbiota homeostasis.

As shown in Fig. 8B, a radar chart with three axes (enterobacteria, lactobacilli and anaerobes) showed the aforementioned modifications of the total colon microbiota in mice with an intestinal inflammatory process fed on a different diet.

## Discussion

The clinical management of patients with intestinal inflammation through completely safe and effective therapies is still an unmet need^24^. Although acid suppressants such as proton pump inhibitors (PPIs) and histamine H_2_-receptor antagonists prevent NSAID-induced damages to the upper GI tract, these agents are not effective at preventing the small intestinal injury^25^. There are evidences that PPIs exacerbated NSAIDs-induced small intestinal damages^26,27^. Even more importantly, as compared with upper GI tract events requiring hospitalization, small intestine cases were associated with higher rates of mortality and relapse, and significantly longer periods of hospitalization^25^.

Understanding the mucosal immune mechanisms in states of health and intestinal disease provides the basis for the development of effective therapeutics aimed at resolving inflammation and preserving intestinal homeostasis. As several studies suggest that intestinal inflammation may be the consequence of a primary deficiency of innate and adaptive immunity^28^, probiotics that are able to reinforce the complex intestinal immune system emerge as promising tools. Although some authors have reported the preventive effect of probiotics in disorders of the gastrointestinal tract by reducing damage in the small intestine, neutrophil infiltration, TNF-α and INF-γ levels^29–31^, the mechanism by which they behave would not be fully elucidated.

Here we focus on the ability of *L. casei* CRL 431 and *L. paracasei* CNCM I- 1518 to preserve intestinal homeostasis in an intestinal inflammatory process induced by indomethacin.

We observed that as intestinal inflammation was elicited by an indomethacin subcutaneous injection in experimental mice, the typical physical and clinical mice aspects were altered, which was reflected by significant weight loss, anemia, piloerection and swelling of the animals (Fig. 1). Additionally, as an indicator of the inflammatory process elicited in mice both the small and large intestine got shorter (Fig. 2). This inflammatory process seems to have caused excessive regenerative hyperplasia of the mucosa leading to a marked thickening of the intestinal wall and the onset of ulcers in the Indo animals receiving a conventional diet (Fig. 2).

Moreover, considering that histomorphology remains a powerful routine tool to evaluate intestinal inflammation, an exhaustive evaluation of the small intestine of the animals was conducted, revealing important alterations of the mucosal architecture. Irregular villi with different heights and widths, enlarged crypts of Lieberkühn and a marked inflammatory cell infiltration were observed in indomethacin-treated animals on a conventional diet (Fig. 3). This infiltration of inflammatory cells in the mucosa further damages the intestinal tissue, leading to a vicious circle. By contrast, the histological index determined for mice receiving the indomethacin injections upon the oral supplementation with *L. casei* CRL 431 or *L. paracasei* CNCM I- 1518 was similar to the healthy controls (Fig. 3). No significant damage in the shape of the intestinal tissue was observed in those animals with respect to controls. Our results suggest that probiotics administered during an intestinal inflammatory process reduce intestinal inflammation through a reinforcement of the intestinal epithelial barrier, particularly, through an increase in the cells responsible for mucus and AMPs production, Goblet and Paneth cells, respectively (Fig. 4).

The role of constitutive and inducible antimicrobial peptides in intestinal inflammation has been investigated thoroughly over the recent years^6,32–35^. Accordingly, a reduced antimicrobial activity of the intestinal fluids of mice with intestinal inflammation was observed against pathogens that usually cause gastrointestinal infections or food poisoning (Fig. 5). By contrast, the CFU/ml of S. Typhimurium and *S. aureus* was significantly reduced after the incubation of these pathogens with the intestinal fluids from indomethacin-treated mice fed with probiotics compared to those on a conventional diet (Fig. 5), reaching counts similar to those observed in the presence of healthy control fluids.

Some antibiotics are effective in preventing damage to the small intestine induced by NSAIDs^36^. However, NSAIDs are frequently prescribed to control chronic pain for a prolonged period, and long-term use of antibiotics increases the risk of eliminating resident microbiota, inducing antibiotic-resident species, creating dysbiosis and developing multi-drug resistant bacteria and enteritis associated with microbial replacement^37,38^. Keeping this in mind, the intestinal antimicrobial activity was tested against multi-drug resistant bacteria. Interestingly, indomethacin-treated mice receiving oral probiotic supplementation displayed an important reduction in the CFU/ml of *Klebsiella pneumoniae* and *E. aerogenes* multi-resistant (Fig. 5) compared to those animals on a conventional diet receiving the indomethacin injection. These results clearly highlight probiotic consumption as an effective therapeutic alternative to many traditional antibiotics due to its lower possibility of generating resistance mechanisms compared to antibiotics, as well as for the safety of its use, as they are endogenous host peptides.

No less important is the fact that *L. casei* CRL 431 and *L. paracasei* CNCM I-1518 administered in our intestinal inflammatory model were also able to modulate the aberrant local and systemic immunity extensively reported in IBD by dampening proinflammatory pathways (Figs. 6 and 7). Furthermore, IL-10, the critical immunoregulatory cytokine in maintaining intestinal homeostasis and symbiosis with enteric microbiota, significantly increases upon probiotic lactobacilli interventions compared to animals with intestinal inflammation receiving a conventional diet (Fig. 6). These results became more relevant after Scheinin et al.’s finding that anti-TNF antibody therapy starting at 4 weeks markedly ameliorated the disease^39^. Moreover, children affected by mutations in the IL-10 receptor are more likely to suffer from very early-onset of IBD^40^. Recently, therapies based on restoring anti-inflammatory signals, by exploiting the tolerogenic potential of cytokines (interleukin-10, transforming growth factor-β, granulocyte macrophage colony-stimulating factor), immune cells (regulatory T cells, tolerogenic dendritic cells), or mesenchymal stem cells, have emerge as effective alternatives with fewer side effects^41^.

A more recent therapeutic strategy for IBD targets on oxidative stress^8^. During mucosal inflammation, intestinal epithelial cells (IECs) as well as neutrophils and macrophages produce superoxide and nitric oxide. These ROS damage cytoskeleton proteins and lead to alterations in tight junctions and epithelial permeability in IECs which in term result in barrier disruption^42^. We postulate that the inflammation initiated by the high levels of ROS and NO production in our model of intestinal inflammation finally causes mucosal injuries accompanied by alterations in IECs, such as Goblet cell loss, crypt cell hyperplasia, and ulceration (Figs. 2, 3 and 4). Notably, the consumption of Lc 431 and Lp 1518 during an intestinal inflammatory process, through a reduction in local secretion of proinflammatory cytokines decreases ROS and NO production by peritoneal macrophages (Fig. 7) and preserves the integrity of the intestinal epithelial barrier.

A still unanswered question is whether the observed microbial dysbiosis is a cause or a consequence of intestinal inflammation. Unquestionably, while in a healthy intestine the Firmicutes and Bacteroidetes phyla predominate and contribute to the production of epithelial metabolic substrates, in IBD patients the microbiota is characterized by a relative lack of these filum, and an over-representation of Enterobacteriaceae and Fusobacteria^43,44^. Moreover, a significantly reduced biodiversity in the fecal microbiome of IBD patients compared to that in healthy controls has also been reported^45,46^. Interesting, the presence of a dysbiotic microbiota that appears after antibiotic treatment, imprints colonic invariant natural killer T (iNKT) and CD4 + T cells toward a pro-inflammatory phenotype that contributes to aggravate intestinal inflammation^38^. Considering those facts, our results in which probiotic consumption modulates the intestinal microbiota in an intestinal inflammatory process by increasing the lactobacilli and anaerobes and reducing the enterobacteria become more relevant (Fig. 8). This beneficial microbiota would be involved in several protective, structural, and metabolic functions that prevent the initiation of an inflammatory process and plays a pivotal role in gut homeostasis.

These results together place *L. casei* CRL 431 and *L. paracasei* CNCM I-1518 at the cutting edge of the novel strategies that are able to reduce intestinal inflammation. Restoring impaired regulatory immune cell activity by correcting dysbiosis and defective antimicrobial activity is a safe and highly promising therapeutic approach to managing an intestinal inflammatory process in a more physiological, safer and sustained manner.

Probiotics emerge as a promising cost-effective therapeutic and preventive strategy to keep intestinal health in patients on non-steroidal anti-inflammatory drug treatment. Probiotics reinforce the intestinal epithelial barrier and preserve intestinal homeostasis targeting several points, namely, Goblet and Paneth cells, antimicrobial activity of the intestinal fluids, balance between pro-inflammatory/regulatory cytokines, oxidative stress-related signaling pathways, and the intestinal microbiota.

## Materials and methods

### Bacteria

*Lactobacillus casei* CRL 431 from the CERELA culture collection and *Lactobacillus paracasei* CNCM I-1518 provided by DANONE Argentina were used as probiotic bacteria. These strains were activated by incubation for 16 h at 37 °C in sterile Man-Rogosa-Sharpe (MRS) broth (Britania, Argentina).

*Salmonella enterica* serovar Typhimurium was obtained from the Bacteriology Department, Hospital del Niño Jesús, Tucuman, Argentina. *Staphylococcus aureus* ATCC 25923, and two human isolated multi-drug resistant bacteria: *Enterobacter aerogenes* (6627734453176010, resistant to: ampicillin, ampicillin/sulbactam, piperacillin/tazobactam, cephalotin, cefotaxime, ceftazidime, cefepime, imipenem, meropenem, nalidixic acid, ciprofloxacin; susceptible to: amikacin, gentamicin, colistin and trimethoprim/sulfamethoxazole), and *Klebsiella pneumoniae* (resistant to: ampicillin ampicillin/sulbactam, piperacillin/tazobactam, cephalotin, cefotaxime, ceftazidime, cefepime, imipenem, meropenem, amikacin, gentamicin, nalidixic acid, ciprofloxacin, nitrofurantoin, trimethoprim/sulfamethoxazole; susceptible to: colistin, were kindly provided by Mariel Cáceres, Bacteriology Laboratory, Hospital Ángel C. Padilla, Tucumán. Aliquots of these bacteria from an overnight culture were placed in 5 ml of sterile Brain Heart Infusion (BHI) broth and incubated at 37 °C to reach exponential grown phase (DO_600_ nm = 0.6).

### Experimental animals and diet

Six to eight weeks old BALB/C mice provided by CERELA-CONICET (Tucumán, Argentina) were used. Animals were maintained in a room with a 12-h light/dark cycle at 22 ± 2 °C and fed with a balanced commercial diet ad libitum.

Lactobacillus bacteria overnight cultures were grown at 37° C in 5 ml sterile MRS broth. The cells were harvested by centrifugation at 5000 g for 10 min, washed three times with phosphate saline solution (PBS) and finally resuspended in 10% non-fat milk. The final concentration of probiotic bacteria was 1 × 10^8^ CFU/ml, a concentration extensively used by our group^47,48^. Bacterial suspensions were diluted 1:30 in water and administered ad libitum to the mice. The suspension was renewed every day and administered for 7 days to groups fed with *L. casei* CRL 431 and for 5 days to mice fed with *L. paracasei* CNCM I-1518. These have been shown to be the periods required for an optimal activation of the intestinal immune system for each strain^49,50^.

BALB/C mice (6 per group) were supplemented with: *Lactobacillus casei* CRL 431 (Lc 431), *Lactobacillus paracasei* CNCM I-1518 (Lp 1518), or water, for 7 and 5 days, respectively. In these animals an intestinal inflammation was induced by two subcutaneous injections of 7.5 mg/kg/day of indomethacin^51^) on the last two days of the experiment protocol. Groups were as follows: Lc 431 + Indo; Lp1518 + Indo; and Indo (control of inflammation).

Additional controls, fed with Lc 431, Lp 1518 or water, respectively, received 2 injection of PBS (Lc 431; Lp 1518 and healthy controls, respectively). The weight of the animals was recorded every two days. Fur appearance of each mouse was analyzed, according to Shrum Bradly^52^. On day 8, mice were euthanized by cervical dislocation after been anesthetized with ketamine-xylazine (80 mg/kg and 10 mg/kg, respectively). Samples of blood, intestinal fluids, large and small intestine, as well as liver and spleen were taken, and their weight and length registered.

A red blood cell test was performed in a Cell-Dyn Ruby analyzer (Abbott) on blood samples taken from experimental mice on the day of their sacrifice.

All animal protocols were preapproved by the Animal Protection Committee of CERELA (CRL-BIOT-LI-2017/3B) and conducted in accordance with the guidelines established by the Consejo Nacional de Investigaciones Científicas y Técnicas (CONICET) and the NHI^53,54^.

### Histological studies

Small intestine samples fixed in a 10% PBS-formaldehyde solution, pH = 7, for 24 h, were then dehydrated at increasing ethanol concentrations (30 to 100%), including in paraffin, sectioned in 4 micron slices, colored with hematoxylin–eosin and examined by light microscopy. The severity of intestinal inflammation was graded from 0 to 4: 0, no leukocyte infiltration; 1, low level of leukocytic infiltration; 2, moderate level of leukocytic infiltration; 3, high level of leukocytic infiltration, and 4, transmural infiltration^55^. A blind histological test of the small intestine was performed, analyzing 100 intestinal crypts in 5 slices of each organ. H&E stain allows the identification of Paneth cells based on their distinctive granule staining pattern. Additionally, villus length and number of Goblet cells in each tissue were analyzed.

### Analyses of large intestine microbiota

Large intestines were removed in aseptic conditions, weighed and placed in tubes containing 5 ml of peptone water (0.1%). Then the samples were homogenized using a tissue microhomogenizer (MSE, England). Serial dilutions were performed and aliquots were spread on the surface of different agarized media: Reinforced Clostridial Agar (RCA) for total anaerobic bacteria, MRS for total Lactobacilli and MacConkey for total enterobacteria. MRS y MacConkey were aerobically incubated at 37 °C for 48 and 24 h respectively; RCA was anaerobically incubated at 37 °C for 120 h.

### Analyses of bacterial translocation

Liver and spleen were removed, weighed and placed in tubes containing 3 ml of peptone water (0.1%). Then the organs were homogenized and aliquots were spread on the surface of MRS and MacConkey media, in pure state and half dilution. Both media were aerobically incubated at 37 °C for 24 h for MacConkey and 48 h for MRS.

### Ex vivo analyses of the antimicrobial activity

The antimicrobial activity from the intestinal fluids of mice were determined as described in Cazorla^23^. Briefly, the small intestines were removed and their content was collected by the passage of 0.5 ml of 10 mM sodium phosphate buffer, pH = 7.4 along the intestine. Intestinal fluids were centrifuged at 1300 xg/4 °C for 15 min and the supernatants incubated in the presence of the pathogenic bacteria. Exponential growth suspensions of *S.* Thyphimurium*, S. aureus*, *E. aerogenes*, *K. pneumoniae* were washed twice in 10 mM sodium phosphate buffer and 10 µl (5 × 10^6^ UFC) of each strain was incubated at 37 °C for 2 h in the presence of 50 µl of intestinal fluids. Serial dilutions of these mixtures were spread in duplicate in BHI agar and incubated at 37 °C for 24 h for CFU count determination.

### Cytokine determination

Intestines and intestinal fluids were collected from mice. Then, intestinal epithelial cells (IECs) were isolated according to Canali^56^ with some modifications. Briefly, small intestines were removed and washed twice in cold PBS. After discarding all Peyer Patches, the small intestines were opened longitudinally, washed with 100 µg/ml gentamicin in PBS, and then, incubated with 8% FBS, 100 µg/ml gentamicin, 1 mM dithiothreitol, 30 mM EDTA in PBS for 15 min at room temperature. Segments were then incubated by shaking, with 100 µg/ml gentamicin, 10 mM EDTA in RPMI at 37 °C for 15 min. Supernatant was collected and centrifuged for 5 min at 300 × g. The pellet was washed twice with RPMI 100 µg/ml gentamicin and finally adjusted to 1 × 10^6^ IEC/ml. Cells were transferred to six-well plates and incubated for 24 h at 37 °C 5% CO_2_. Supernatants were recovered for cytokine determination.

TNF-α and IL-10 were determined by enzyme-linked immunosorbent assay according to the manufacturer’s instructions (BD OptEIA; BD Biosciences, USA).

### Enzyme markers of inflammatory process and tissue damage

Levels of C-reactive protein (CRP) and lactate dehydrogenase (LDH) were analyzed in mice serum, following instructions from the commercial kits (GT Lab, Argentina).

### Ex vivo determination of ROS and NO production

Cellular production of ROS was analyzed using H2DCFDA-related labelling. H2DCFDA (Sigma), an uncharged and non-fluorescent compound, freely diffuses across membranes in cells where it is hydrolyzed by esterase, and further oxidized by ROS into dichlorofluorescein (DCF), a strong green fluorescent substance that cannot pass through the cell membrane. Cells (1 × 10^6^) isolated from murine peritoneal cavity were washed with PBS, and incubated with 10 µmol/l H2DCFDA at 37 °C for 1 h in the dark, then washed and resuspended in PBS. Cellular H2DCFDA-related fluorescence that reflects ROS formation was thereafter measured by flow cytometry using a BD FACSCalibur TM flow cytometer (BD Biosciences), and data were analyzed using Flow Jo software (Tree Star).

Additionally, 1 × 10^6^ cells isolated from the murine peritoneal cavity were seeded in a 24-well plate and incubated for 18 h at 37 °C 5% CO_2_. Later, supernatants were removed and stored at -80 °C for cytokine and nitric oxide (NO) determination. Cytokines were determined using the corresponding enzyme-linked immunosorbent assay set according to the manufacturer’s instructions (BD OptEIA; BD Biosciences, USA).

The amount of NO released into the culture medium by macrophage cells was measured using Griess reagent. The reaction products were measured in a spectrophotometer at 550 nm, and their concentration was subsequently determined on a curve constructed with known concentrations of NaNO_2_.

### Statistical analyses

Results are presented as means ± SEM. GraphPad Prism 5.0 software (GraphPad Software Inc., San Diego, CA) was employed to carry out calculations. Results presented are representative of three independent experiments. The statistical significance was determined by one-way analysis of variance (ANOVA), using the Kruskal–Wallis test^57^. *P* values of < 0.05 were considered to be statistically significant.

The radar chart tool in Microsoft Excel 2016 was used for a comparative study. Results were expressed as the relation of the experimental and healthy control animals.
